# Sleep, Circadian Rhythmicity and Response to Chronotherapy in University Students: Tips from Chronobiology Practicals

**DOI:** 10.5334/jcr.202

**Published:** 2021-01-21

**Authors:** Sara Montagnese, Lisa Zarantonello, Chiara Formentin, Christian Zancato, Maria Beatrice Bonetto, Alberto Biscontin, Paola Cusumano, Rodolfo Costa

**Affiliations:** 1 Department of Medicine, University of Padova, Padova, Italy; 2 Department of Biology, University of Padova, Padova, Italy

**Keywords:** chronobiology, sleep timing, temperature, chronotherapy, students, teaching

## Abstract

Chronobiology is not routinely taught to biology or medical students in most European countries. Here we present the results of the chronobiology practicals of a group of students of the University of Padova, with a view to highlight some interesting features of this group, and to share a potentially interesting cross-faculty teaching experience. Thirty-eight students (17 males; 22.9 ± 1.6 yrs) completed a set of self-administered electronic sleep quality [Pittsburgh Sleep Quality Index (PSQI)], chronotype and sleepiness [Epworth Sleepiness Scale (ESS)] questionnaires. They then went on to complete sleep diaries for two weeks. Sixteen also wore an actigraph, 8 wore wireless sensors for skin temperature, and 8 underwent a course of chronotherapy aimed at anticipating their sleep-wake timing. Analyses were performed as practicals, together with the students. Average PSQI score was 5.4 ± 1.9, with 15 (39%) students being poor sleepers. Average ESS score was 6.5 ± 3.3, with 3 (8%) students exhibiting excessive daytime sleepiness. Seven classified themselves as definitely/moderately morning, 25 as intermediates, 6 as moderately/definitely evening. Students went to bed/fell asleep significantly later on weekends, it took them less to fall asleep and they woke up/got up significantly later. Diary-reported sleep onset time coincided with the expected decrease in proximal skin temperature. Finally, during chronotherapy they took significantly less time to fall asleep. In conclusion, significant abnormalities in the sleep-wake patterns of a small group of university students were observed, and the students seemed to benefit from chronotherapy. We had a positive impression of our teaching experience, and the chronobiology courses obtained excellent student feedback.

## INTRODUCTION

In spite of its increasing relevance to both basic science and medicine [1], chronobiology is not routinely taught to biology or medical students in most European countries, except for anecdotal experiences [2]. These include autorhythmometry (i.e. students play the role of participants and experimenters), which seems to be a useful method for students to both understand chronobiological concepts [2] and to develop healthy sleep habits [3]. In further detail, Rol de Lama and co-workers [3] collected two weeks of sleep-wake diaries, sleep quality, and the acrophase of different physiological and laboratory parameters from medical students. After analysing their own data, students reported more awareness about the influence of the acrophase. In addition, they were more compliant with course lessons attendance, and performed better in the exam. Similarly, Azevedo and colleagues [2] developed a high school sleep educational program which led not only to increased knowledge but also to changes in sleep habits, with the students exhibiting a reduction in naps duration and in the discrepancy between bedtimes in the week and at the weekend.

Two of us (RC and SM) have been running chronobiology courses for several years for both molecular biology students (within the neurobiology modules) and for medical students (non-compulsory chosen modules) at the University of Padova, in Italy. Both courses are run during the second semester (March-May) for two to four hours per week, and they have partly overlapping programmes, with molecular biology students learning more about the neurogenetic bases of circadian rhythmicity and the functional organization of the biological clock [4], and medical students learning more about the mechanisms of clock synchronisation and the health consequences of desynchronization [5,6]. Over the years practicals, which also have partly overlapping programmes, have included genotyping for clock gene polymorphisms [7], definition of chronotype [8], and direct experience of the effects of light administration on sleep-wake patterns and melatonin urinary metabolites [9,10]. Students from both courses have also been welcome to test actigraphs, polygraphs, skin and core temperature sensors and other tools for the evaluation of circadian rhythmicity, in either of the laboratories run by RC and SM.

Here we present the results of the practicals of a group of students (academic year 2018–2019) who all underwent sleep-wake evaluation, and some of whom also underwent actigraphy recordings, 24-hour skin temperature measurement, and/or a short course of chronotherapy (i.e. light administration in the morning and light shading in the evening/night hours) aimed at anticipating their sleep-wake rhythm. Timed exposure to bright light and/or protection from light can reset the circadian pacemaker [11,12,13] by up to 12 hours in 2–3 days [13,14.]. This technique has already been used in adolescents and young adults with delayed sleep phase type disorder, who showed improvement in sleep timing, sleep onset latency and daytime functioning after three weeks [15]. Other applications of chronotherapy include mood disorders [16], depression [17], dementia [18], and the management of insomnia in both children [19] and older adults [20].

Our manuscript aims at highlighting some interesting features of a small, homogenous population of university students, and also at sharing a somewhat unique and fruitful, cross-faculty teaching experience.

## METHODS

### TEACHING

RC opened SM’s course to medical students, teaching them the basics of genetic and molecular chronobiology for three lessons of two hours each; SM closed RC’s course to molecular biology students teaching them about sleep-wake disturbances and the basics of a clinical circadian assessment for three lessons of two hours each.

### SLEEP-WAKE ASSESSMENT

Having almost reached the end of their course (May 2019, Daylight Saving Time in Italy), 38 students (17 males; 22.9 ± 1.6 years of age; 4 qualifying themselves as shift-workers) completed a set of self-administered electronic (via a link to REDCap electronic data capture tools hosted at the University of Padova [21]) sleep quality, chronotype and sleepiness questionnaires, with instructions being imparted to them as a group, at the same time, by authors SM and LZ. They then went on to complete electronic sleep diaries for a fortnight (13.6 ± 3.4 days) at home, on waking up in the morning. In further detail, they completed:

- The Pittsburgh Sleep Quality Index (PSQI), which evaluates subjective sleep quality over the preceding month, and differentiates “good” from “poor” sleepers. Responses to the 24 questions of this self-administered questionnaire are used to generate seven components, each of which is scored from 0 to 3 (0 = best). The PSQI total score is the sum of all domains (range 0–21), and a total score >5 characterizes “poor sleepers” [22,23].
- The Horne–Östberg (HÖ) questionnaire, which defines chronotype as definitely morning (score 70–86), moderately morning (59–69), intermediate (42–58), moderately evening (31–41), and definitely evening (16–30) based on 19 self-administered questions aimed at identifying preferred time of day for carrying out certain activities [24,25].
- The μ-Munich Chronotype Questionnaire (μ-MCTQ, [26]), a shortened, 6-question, validated version of the MCTQ [27], which allows to assess chronotype by asking subjects about their sleep-wake behaviour in free versus work/study days, and to quickly obtain the variables midlseep (the difference or corrected difference between sleep onset and wake up time, in clock time) and sleep duration.
- The Epworth Sleepiness Scale (ESS), which is used to assess daytime sleepiness. Subjects rate their likelihood of ‘dozing off’ in eight different daytime situations, on a scale of zero (unlikely), to three (very likely). The component scores are summated to provide a total score (range: 0–24); a score of ≥11 is considered abnormal [28,29].
- Daily sleep diaries, recording bedtime, sleep onset, time taken to fall asleep, wake up time, get up time, and the number and duration of any night awakenings and/or daytime naps. Sleep-onset latency was calculated as the difference between bedtime and sleep onset time (in minutes); time spent in bed as the difference between bedtime and get up time (in hours); sleep duration as the difference between sleep onset and wake up time (in hours). Finally, the difference in minutes between wake up time and get up time was also calculated. Each diary page also included a visual-analogue scale for the assessment of sleep quality during the previous night (range 1: worst to 10: best) [30,31]. Diaries were analysed individually, and also averaged by week days versus weekends (i.e. the night between Friday and Saturday and Saturday and Sunday; please also note that these may or may not coincide with work/study days and free days as defined in the μ-MCTQ).

A full sleep-wake report including all questionnaire scores, averaged sleep diary information and a clinical comment, formulated by authors LZ and SM, was produced for each student. Having obtained the students’ permission, reports were then discussed over a subsequent practical session.

### ACTIGRAPHY

While continuing to fill in their sleep-wake diaries, 16 students (6 males; 23.4 ± 1.9 years of age; 1 qualifying themselves as shift-workers) also wore an actigraph (PRO-Diary or MotionWatch 8, Camntech, Cambridge, UK) for 2–3 days, depending on the opportunities they had over the week in between two formal lessons to meet (during other lessons, over common travel routes or on social occasions) and swap actigraphs, having cleaned them according to the instructions received, and recording the exact date/time of the exchange on the pertinent sleep diary of both students involved. Thus the four actigraphs available at the time for the practicals made it possible to obtain 48-hour recordings from 16 students over 8 days. Actigraphy recordings were then analysed visually and automatically, with or without the pertinent sleep diary information being fed into the CamNtech MotionWare Software programme. This was done together with the students, during a subsequent practical session.

### PROXIMAL TEMPERATURE ASSESSMENT

While continuing to fill in their sleep-wake diaries, 8 students (3 males; 22.1 ± 1.3 years of age; 2 qualifying themselves as shift-workers) also wore three wireless skin temperature sensors (iButtons, model no. DS1922L-F5, Maxim Integrated, San Jose, CA, USA) placed on the abdomen, right infra-clavicular area and left mid-thigh (as detailed in [32]) for 24 hours. Sampling rate was set at 3 minutes (resolution 0.0625°C; approximately 500 temperature values per day). Data from the sensors were transferred by an adapter to a computer, using the iButton Viewer software (Dallas Semiconductor, Maxim Integrated Products, Sunnyvale, CA, USA). An artefact rejection procedure was applied to exclude extremes. Proximal skin temperature was then calculated as detailed in [32]. Recordings were analysed visually, together with the students, and sleep onset times from the corresponding diaries added to the temperature plots, with a view to identify the decrease in proximal temperature that normally precedes sleep onset [33].

### CHRONOTHERAPY TRIAL

**Figure 1:**
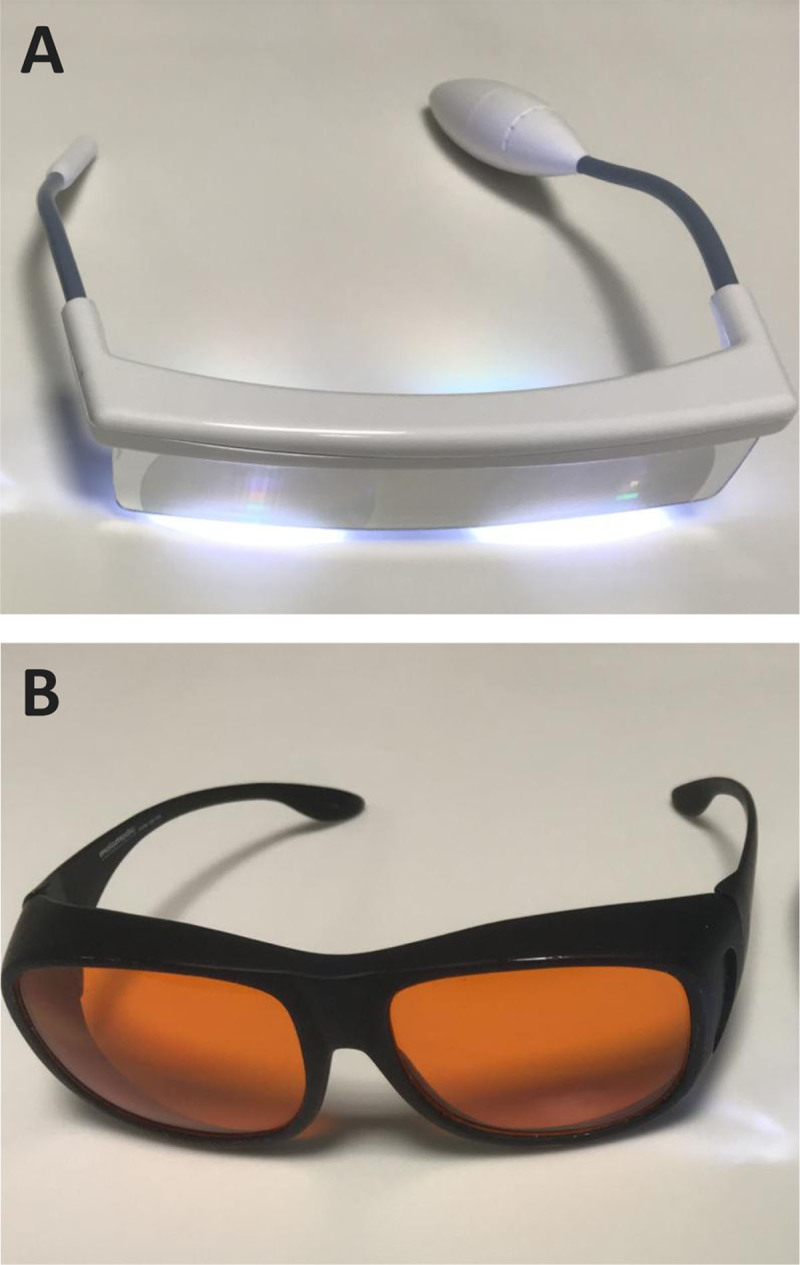
Luminette light glasses [Lucimed, Villers-le-Bouillet, Belgium; (**A**)] and short-wavelength filter glasses [MelaMedic, Viborg, Denmark; (**B**)].

While continuing to fill in their sleep-wake diaries, 8 students (5 males; 23.6 ± 1.6 years of age; 1 qualifying themselves as a shift-worker) underwent a chronotherapy trial aimed at anticipating their sleep-wake rhythm for 5–8 days, depending on both tools availability and students’ own routines. They were provided with light glasses (***Figure 1A***) and short-wavelength filter glasses (***Figure 1B***), and instructed to wear the light glasses for 30 min in the morning, after waking up (i.e., while having breakfast, immediately after their personal hygiene etc.) and the short-wavelength filter glasses in the evening, for the two hours prior to bedtime, plus at any time overnight when the light in their room was on/they used the bathroom etc.

Luminette R light glasses [34] (Lucimed, Villers-le-Bouillet, Belgium; ***Figure 1A***), which can be worn over prescription glasses, are equipped with eight Light Emitting Diodes (LEDs) distributed on the upper part of the lenses, outside the patient’s visual field. The LEDs reflect light (2000 lux; blue-enriched 400–750 nm) towards the eye via a diffractive lens, thus focusing it towards the lower part of the retina, regardless of the position of the head [35]. Short-wavelength filter glasses (MelaMedic, Viborg, Denmark; ***Figure 1B***), which can also be worn over prescription glasses, filter light in the blue range of the spectrum, i.e. the one our visual circadian timing system is most sensitive to [36]. Thus they limit exposure to awakening light signals at times when the natural environment is dark but the artificial one may be well lit or the subject may be using light-emitting devices such as computers, mobile phones, tablets etc. Diary-derived sleep timing parameters were averaged prior to (7.3 ± 1.9 days) and during the chronotherapy trial (6.1 ± 1.5 days).

### ETHICS

Prior to commencement of the practicals, permission was obtained from the Ethics committee of Padova University Hospital in the form of an amendment to protocol 3639/AO/15, and subsequent modifications.

### STATISTICAL ANALYSES

The distribution of variables was tested for normality using the Shapiro–Wilk’s W test and the data are presented as mean ± SD or median and range, as appropriate. Differences between normally and non-normally distributed variables were tested by the Student t or the Mann Whitney U test (post hoc: Bonferroni), respectively. Data from the four shift workers were analyzed both together and separately from the rest of the students, as appropriate. Repeated measures ANOVA or the Wald test were used to compare pre and treatment sleep timing variables, taking into account of time of the week (week days and weekends). Categorical variables were compared by the Pearson’s χ^2^ or Fisher’s exact test, as appropriate. Analyses were carried out by the programme R, Version 1.2.5033 (R Core Team 2017) and the nparLD package [37] was used to perform the Wald test.

## RESULTS

Average PSQI score was 5.4 ± 1.9, with 15 (39%) students being qualified as poor sleepers. Average ESS score was 6.5 ± 3.3, with 3 (8%) students exhibiting excessive daytime sleepiness. Based on the HÖ questionnaire, one student (3%) qualified themselves as being definitely morning, 6 (16%) as moderately morning, 25 (66%) as intermediates, 5 (13%) as moderately evening and 1 (3%) as definitely evening. Based on the μ-MCTQ, midsleep was 03:24 ± 00:42 on work/study days and 04:54 ± 00:54 on free days; corrected midsleep was 04:12 ± 00:54. Females woke up earlier than males on both week days and weekends (07:00 ± 00:30 vs. 07:30 ± 00:42, p < 0.01; 07:42 ± 01:12 vs. 08:24 ± 00:54, p < 0.05), and also got up earlier on week days (07:24 ± 00:30 vs. 07:54 ± 00:48, p < 0.05); no differences were observed between females and males in any of the other sleep-wake variables.

Average sleep timing parameters obtained from sleep diaries on week days and weekends are presented in ***Table 1***. Students went to bed and fell asleep significantly later on weekends, it took them less to fall asleep, they woke up and they got up significantly later and they also spent significantly more time in bed after waking up. Finally, fewer students [(11/38 (29%) vs. 20/38 (53%)] napped on weekends, and weekend naps were significantly shorter (***Table 1***).

**Table 1:** Mean ± SD (range) diary-derived sleep timing variables, on week days and weekends.


	WEEK DAYS	WEEKENDS

**Bed time (clock time)**	23:36 ± 01:00 (21:42–02:42)	00:18 ± 00:48 (22:00–01:30) °°°

**Sleep onset latency (min)**	13 ± 10 (1–48)	11 ± 7 (1–35) °°°

**Sleep onset time (clock time)**	00:18 ± 00:54 (22:30–03:12)	00:48 ± 00:48 (22:30–02:12) °°°

**Awakenings (n)**	0.6 ± 0.5 (0.0–2.0)	0.6 ± 0.7 (0.0–2.7)

**Time spent awake (min)**	12 ± 19 (1–110)	12 ± 11 (2–45)

**Wake up time (clock time)**	07:12 ± 00:36 (05:54–08:42)	08:00 ± 01:06 (03:30–10:18) °°

**Get up time (clock time)**	07:36 ± 00:42 (06:36–09:42)	08:36 ± 00:54 (06:24–10:36) °°°

**Difference wake-get up time (min)**	22 ± 18 (2–91)	38 ± 35 (0–178) °°°

**Sleep duration (h)**	6.9 ± 0.1 (4.1–8.1)	7.2 ± 0.1 (5.0–8.9) ^§^

**Subjective sleep quality (1–10)**	6.2 ± 1.2 (4.0–9.0)	6.2 ± 1.3 (3.3–9.0)

*Naps (n)	0.5 ± 0.5 (0.0–1.0)	0.3 ± 0.4 (0.0–1.0)

*Nap onset time (clock time)	15:42 ± 01:36 (14:12–20:00)	14:06 ± 02:18 (07:48–16:54)

*Nap offset time (clock time)	16:36 ± 01:48 (14:42–21:18)	15:00 ± 02:30 (08:24–18:12)

*Nap length (min)	50 ± 28 (15–105)	46 ± 37 (10–115) °


* n = 20 for week days and n = 11 for weekends. Significance of the difference between week days and weekends: ^§^ 0.05 < p < 0.1 (trend), ° p < 0.05, °° p < 0.01, °°° p < 0.001. *Note*: Significance levels remained unchanged or improved when analyses were repeated having removed the student with recent intercontinental travel, whose diaries are also responsible for most of the extremes observed in the ranges.

**Figure 2:**
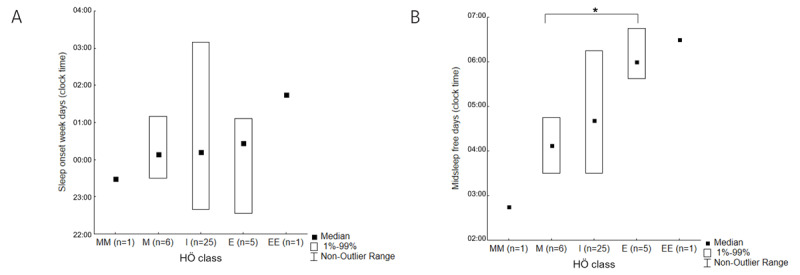
Diary-derived sleep onset time on week days (**A**; p = 0.4, n.s.) and midsleep on free days (**B**; p = 0.0022; * p = 0.008 on *post-hoc* analysis) by Horne–Östberg class, with MM meaning definitely morning, M moderately morning, I intermediate, E moderately evening, and EE definitely evening.

Given the low numbers, no significant differences in sleep timing were observed by splitting students into HÖ diurnal preference/chronotype categories; however, trends were obvious (***Figure 2A***). By contrast, significant differences in free days midsleep (***Figure 2B***) and corrected midsleep were observed by splitting students into HÖ diurnal preference/chronotype categories. Average, summary sleep timing parameters as declared in the μ-MCTQ were also consistent with more punctual daily diary entries, despite the fact that diaries were split by weekdays versus weekends while the μ-MCTQ compares work/study and free days. Students with abnormal PSQI score had significantly more overnight awakenings (0.9 ± 0.1 vs. 0.3 ± 0.1, p < 0.05) and spent significantly more time in bed after waking up (44 ± 7 vs. 22 ± 6 min, p < 0.05). Students with abnormal ESS went to bed significantly earlier than those with normal ESS on weekends (22:06 ± 00:54 vs. 00:24 ± 00:48, p < 0.01). Students who qualified themselves as shift workers went to bed and fell asleep later on week days (00:30 ± 01:36 vs. 23:30 ± 00:54, p < 0.05; 01:06 ± 01:24 vs. 00:06 ± 00:42, p < 0.05), and reported significantly worse subjective sleep quality on both week days and weekends (5.0 ± 1.2 vs. 6.3 ± 1.1, p < 0.05; 4.7 ± 1.4 vs. 6.7 ± 1.5, p < 0.05).

**Figure 3:**
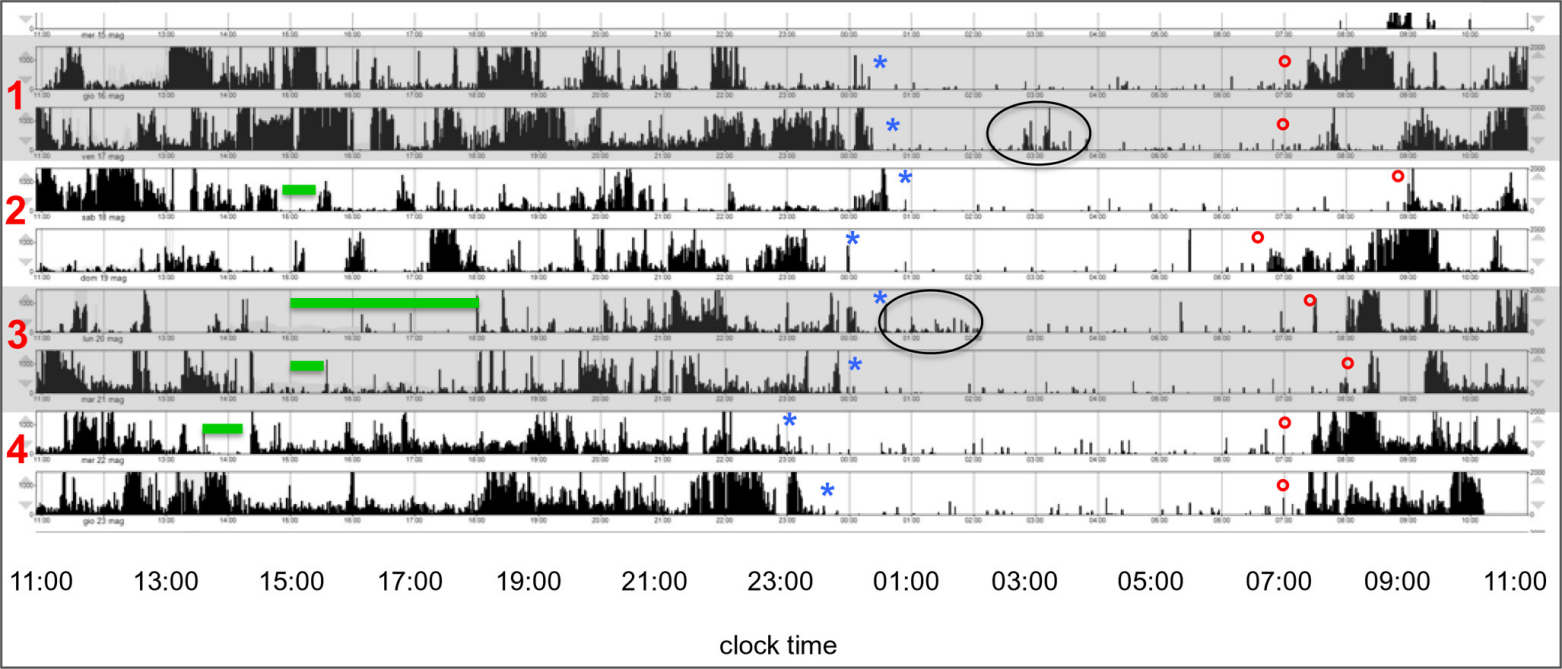
48-hour actigraphy plots of four students [**student 1:** female, 23 yrs, moderately morning, Pittsburgh Sleep quality Index (PSQI) normal, Epworth Sleepiness Scale (ESS) normal; **student 2:** female, 24 yrs, intermediate, PSQI abnormal, ESS normal; **student 3:** female, yrs, intermediate, PSQI normal, ESS normal; **student 4:** female, yrs, intermediate, PSQI normal, ESS normal] marked alternatively in grey and white. Sleep onset times from the corresponding sleep diary are marked as blues asterisks, wake up times as red circles, and naps as green lines. The two black circles mark a night awakening in Student 1 and a slightly disturbed sleep onset in Student 3.

As students exchanged actigraphs every 2–3 days, four actigraphy plots were obtained from 16 students, each with information from four students. One such plot is presented as an example in ***Figure 3***. Demographic and summary sleep-wake details of each student are indicated in the legend, while sleep onset time, wake up time and naps, as reported on the corresponding sleep diary, are marked on the plot. Some heterogeneity can be observed in the activity profiles both within (Student 2 on days 1 and 2, which were a Saturday and a Sunday) and across subjects (Students 1 and 3). At least one prolonged nocturnal awakening (Student 1, day 2) and a slightly disturbed sleep onset (Student 3, day 1) can be observed.

**Figure 4:**
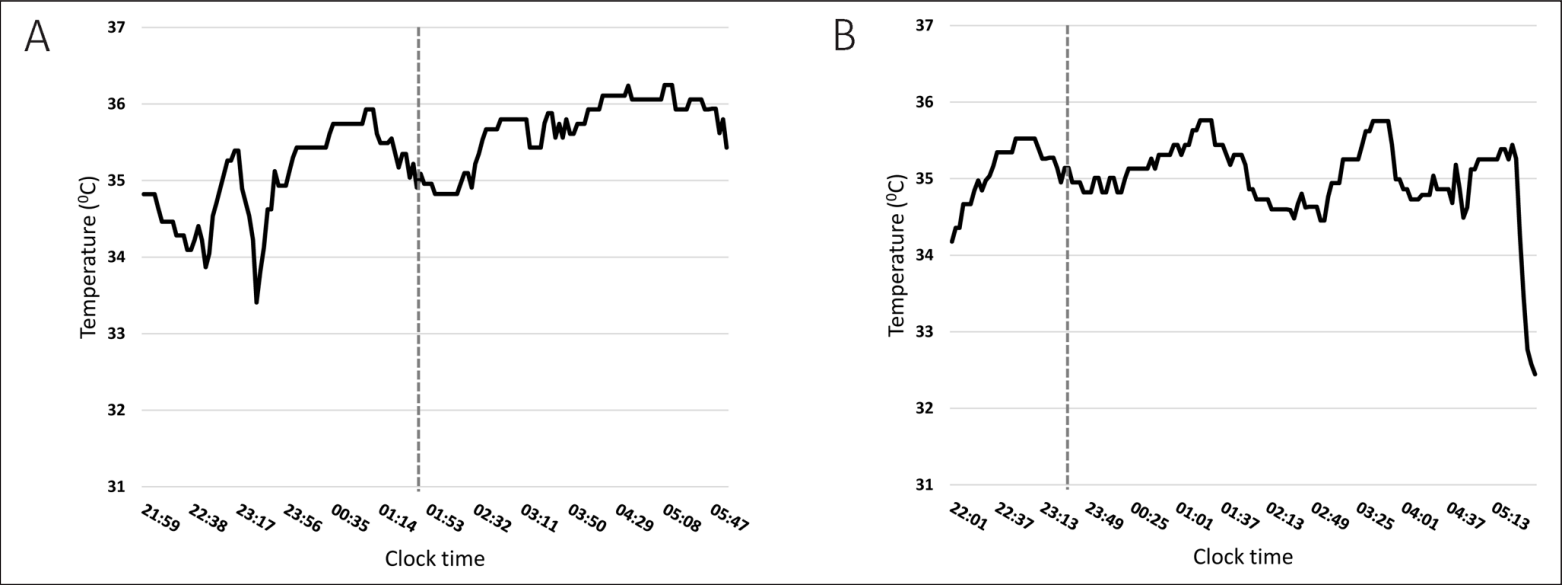
Proximal skin temperature over time (from 22:00 to 06:00 hours) in a 22-year old female student (**A**) and a 23-year old female student (**B**) whose sleep onset timing on that day (broken vertical line) was 01:35 and 23:30, respectively. In both instances, declared sleep onset timing immediately preceded/accompanied a dip in proximal temperature. The sudden and large decrease in temperature in plot B at around 05:30 was most likely due to displacement of one of the three sensors.

Two example 24-hour temperature plots with superimposed sleep onset timing are presented in ***Figure 4A–B***. In these, as in most other instances (7 out of 8, 87%), reported sleep onset time did actually coincide with a decrease in proximal skin temperature. In the overall group, this was of 0.6 ± 0.4°C (0.0–1.3) from the previous identifiable peak, within 15 minutes of time.

**Figure 5:**
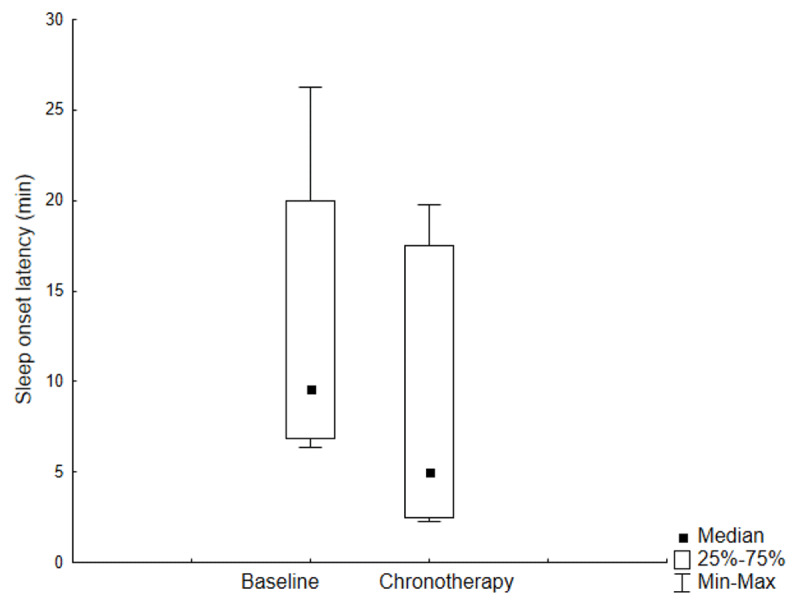
Diary-derived sleep onset latency, in minutes, prior to and during the course of chronotherapy (p < 0.05), adjusted for time of the week.

**Figure 6:**
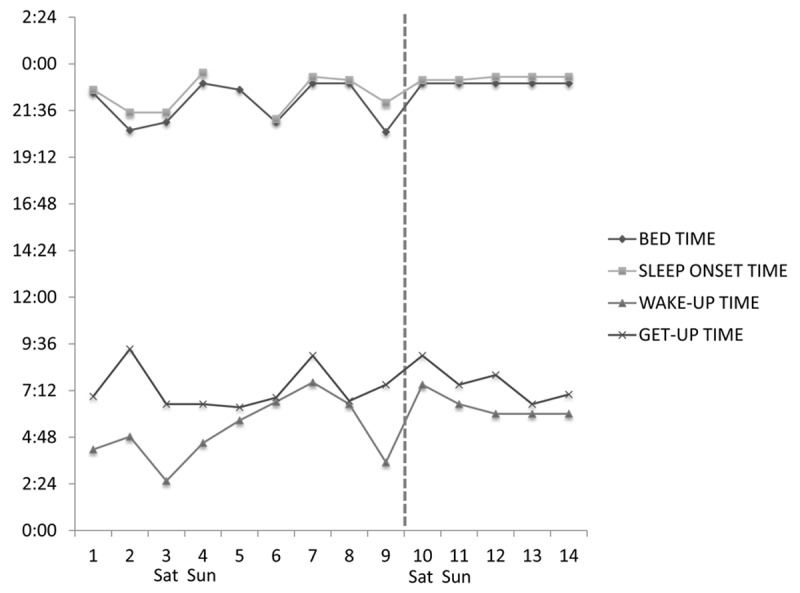
Bed time, sleep onset time, wake up time and get up time over the course of 14 days in the 23-year old female student who had recently returned from Asia. The commencement of chronotherapy (broken vertical line) seemed to regularize her sleep-wake timing. Saturdays and Sundays are marked on the x axis.

On analysing the chronotherapy trial data, it became evident that one student had had an extremely irregular schedule and it was discovered that she had travelled intercontinentally over 4.5 times zones East; her data were thus removed from the group and analysed separately. Whilst wearing the light and short-wavelength filter glasses, students took significantly less to fall asleep compared to baseline (***Figure 5***), also when time of the week (week days versus weekends) was taken into account. This held true when the shift-working student was also removed. Chronotherapy did not result in changes in any other sleep-wake parameter. Chronotherapy seemed to help adjusting to local time the student who had travelled intercontinentally (***Figure 6***).

## DISCUSSION

Over a third of the students enrolled were poor sleepers (complaining of frequent night awakenings) and just under 10% exhibited excessive daytime sleepiness (going to bed earlier on weekends); sleep-wake timing was quite delayed, as expected within their age range. As observed in other studies, females tended to wake up and get up earlier than males [38,27]. The differences between sleep-wake timing on week days versus weekends and the features of weekend naps (less common and shorter) support the contention that young adults with a tendency towards delayed sleep-wake timing are sleep-deprived during week days; however, the difference in night sleep duration between week days and weekends fell short of statistical significance in this relatively small group. These observations are substantially in line with those of earlier series from our own labs, and also with those of large, comprehensively-characterised groups of university students in other countries [39,40,41]. Albeit short, actigraphy plots seemed to confirm the results of questionnaires and diaries.

Four students (10%) qualified themselves as shift workers; these individuals went to bed and fell asleep later on week days, and reported significantly worse subjective sleep quality. Frequency and general information on shift working amongst students (and/or individuals of their age) may or may not be reliable when based on national statistics, as students’ jobs are often temporary, sometimes shared etc. Nonetheless, the number of implicated students in this group was not negligible, and their sleep clearly worse. Of course, this might depend on a host of reasons, as students who need to work in order to support their studies may have economic worries and/or less comfortable lifestyles, thus reasons other than shift work *per se* to sleep worse than their counterparts who can afford to study without working.

Of great interest, promising results were obtained from a short, locally-developed chronotherapy protocol, based on the combination of light administration in the morning and shading in the evening, which we have previously tested in a pilot study of patients with hospitalisation-related insomnia [42]. Treatment was well tolerated, none of the students reported any side effects, and sleep onset latency decreased significantly. This, considering that the course of treatment was very short and no control whatsoever exercised on compliance, is impressive, especially by comparison with results we have previously obtained in patients [42,43]. Of course, in this instance we treated young healthy adults of an age when: *i)* the retinal-hypothalamic-pineal tract is expected to be unimpaired in anatomy and physiology; *ii)* features of delayed sleep phase disorder are still fairly common, especially in males, and *iii)* poor light-dark hygiene is also common, in relation to the widespread use of light emitting devices such as computers, tablets and smart-phones in the evening and night hours. The light that comes from such devices is relatively strong and blue-enriched, thus the short-wavelength filter glasses might have been particularly useful [44,45]. It would be interesting, in future studies, to test short-wavelength filter glasses as a stand-alone, as the effects observed in this study might depend on either device, or their combination. The effect of chronotherapy in the student who had just come back from Asia was striking, confirming that jet-lag remains an extremely fitting application for chronotherapy, with very well-documented beneficial effects [46].

Finally, proximal temperature was obtained by a validated, reduced number of sensors method we developed a few years back [32] and again, despite the students involved being active and maintaining their daily routines, no problems were encountered in obtaining stable, readable recordings. The expected, tight relationship between a decrease in proximal temperature and declared sleep onset time on the pertinent sleep diary was observed in most instances. These data are also interesting, as they may lead to novel management options for delayed sleep-wake timing in the young. Modulation of proximal and especially distal temperature (by heating/cooling the extremities, which is easier than heating/cooling the abdomen and chest) has already been shown to affect sleep onset latency in controlled laboratory conditions [47] and in primary vasospastic syndrome, a rheumatological disorder characterised by difficulties in vasodilating the extremities, and thus prolonged sleep onset latency [48]. Temperature modulation of the extremities could, of course, be tested also in young individuals with delayed sleep-wake habits and difficulties falling asleep.

The study has several limitations, the main one being that it was unplanned, with no *a priori* power calculations. However, the fact that even the experiments with small sample sizes (for example the chronotherapy course) yielded significant results is interesting and it most likely attests to the students’ interest, participation and rigorous adherence to the instructions received. This is in line with previous observations suggesting that when students are both experimenters and participants to a study, this helps them familiarize with the chronobiological concepts underlying the study [2]. Unfortunately, by contrast with previous studies [3], we did not collect follow up data, and therefore we do not know whether the experience also resulted in healthier sleep-wake habits over time.

The chronobiology courses, and the practicals in particular, obtained excellent student feedback. Several students stayed on to complete their final dissertation projects, and several were supervised across the two labs, helping to complete studies which would have otherwise taken longer and possibly been less fruitful. Over the years, medical students have very much enjoyed the basic chronobiology lessons they received from RC, and also the enthusiasm around the 2017 Nobel prize for Medicine or Physiology being assigned to Professors Hall, Rosbash and Young for their contributions to the understanding of the mechanisms underlying circadian rhythmicity. On the other hand, molecular biology students have had glimpses of clinical chronobiology, and the opportunity to experience the reality of circadian rhythm disorders, and the difficulties in diagnosing and managing them in real patients. Molecular biology and medicine are considered to be closed disciplines, but they are indeed fairly separate worlds, with limited integrated teaching. Indeed, while our experience may at some stage lead to an integrated chronobiology course at the University of Padova, so far it has remained an informal albeit structured collaboration, and it has essentially depended on our shared conviction that medical students need the basics of chronobiology to understand circadian medicine, and molecular biology students need some information on clinical applications to put their knowledge into perspective. We ourselves, unfailingly attending each other’s lessons, have greatly benefitted from them, and from developing our teaching into training through the practicals.

In conclusion, significant abnormalities in the sleep-wake patterns of a small group of university students were observed, together with the expected tendency to delayed sleep-wake timing, which seemed to benefit from chronotherapy. Further, our cross-faculty teaching experience suggests that in this era of translational chronobiology, a mixture of basic science and pragmatic medical application is good for teaching, good for research, and good fun.
